# Supplementary material for: Computational Design of Ni6@Pt1M31 Clusters for Multifunctional Electrocatalysts
Source: Molecules. 2023 Nov 13;28(22):7563. doi: 10.3390/molecules28227563 (PMC10675175; doi:10.3390/molecules28227563)
Supplement: Supplementary file 1 [file molecules-28-07563-s001.zip › molecules-2475250-supplementary.docx]

**Supplementary information**

**for**

**Computational design of Ni_6_@Pt_1_M_31_ clusters for multifunctional electrocatalysts**

Jiaojiao Jia, Dongxu Tian^*^

(*State Key Laboratory of Fine Chemicals, School of Chemical, Dalian University of Technology, Linggong Road, Liaoning 116024, China*)

Text S1

The formation energy of Ni_6_@Pt_1_M_31_ was defined as

$\text{E}_{\text{f}}\text{ = }\text{E}_{\text{Ni6@Pt1M31}}\text{ + }\text{μ}_{\text{M}}\text{ – (}\text{E}_{\text{Ni6@M32}}\text{ + }\text{μ}_{\text{Pt}}\text{)}$ (S1)

where *E*_Ni6@M32_ and *E*_Ni6@Pt1M31_ denote the total energy of Ni_6_@M_32_ and Ni_6_@Pt_1_M_31_, respectively. $\text{μ}$_Pt_ and $\text{μ}$_M_ are the chemical potentials of Pt and M (M= Pd, Cu, Ag, Au) respectively. The chemical potential of M (including Pt) was calculated from optimized geometry of bulk structures. The chemical potential of M indicates dividing the total energy of the bulk structure by the number of metal atoms in the bulk (${}_{M}=E_{\mathrm{bulk}}/n$).

The average binding energy of the clusters and SACs was calculated according to the following equation:

$\text{E}_{\text{b}}\text{= (6}\text{ }\text{× }\text{E}_{\text{Ni}}\text{+}\text{ }\text{32}\text{ }\text{×}\text{ }\text{E}_{\text{s}\text{h}\text{ell}\text{ }}\text{-}E_{catalyst}\text{)/38}$ (S2)

where $\text{E}_{\text{core}}$, $\text{E}_{\text{s}\text{h}\text{ell}\text{ }}$, $E_{catalyst}$ correspond to the total energies of Ni core, M shell and Ni_6_@M_32_ or Ni_6_@Pt_1_M_31_ catalysts. $\text{E}_{\text{core}}$ and $\text{E}_{\text{shell}}$ denotes total energies of Ni atom and M (M= Pt, Pd, Cu, Ag, Au) atom which equal to zero.

The HER mechanism^[^[^1-3^](#_ENREF_1)^]^ in acidic electrolyte for reducing protons to hydrogen includes two possible reaction pathways. the Volmer–Heyrovsky and the Volmer–Tafel mechanism are as follow:


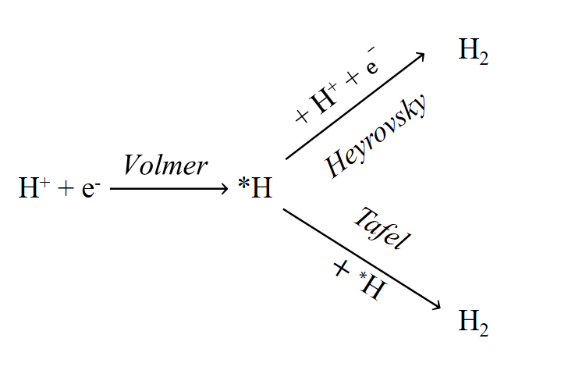


Electrocatalyst performance under the standard conditions was evaluated on the basis of Gibbs free energy for H-adsorption (Δ*G*_H*_), a key parameter for HER activity, following the equation:

${\text{∆}\text{G}}_{\text{H*}}\text{=}{\text{∆}\text{E}}_{\text{H*}}\text{ +}{\text{ ∆}\text{E}}_{\text{ZPE}} \text{- }\text{T}{\text{∆}\text{S}}_{\text{H}}$ (S3)

where ${\text{∆}\text{E}}_{\text{H*}}$ represents the hydrogen adsorption energy, ${\text{ ∆}\text{E}}_{\text{ZPE}}$ is zero-point energy difference between adsorbed hydrogen and gas-phase hydrogen, and the third value ${\text{∆}\text{S}}_{\text{H}}$ represents the entropy difference between adsorbed state and gas phase under the temperature is 298.15K. In order to measure the ${\text{ ∆}\text{E}}_{\text{ZPE}}$ and $\text{T}{\text{∆}\text{S}}_{\text{H}}$, the vibrational frequencies of the system studied should be calculated.

where ∆𝐸_H*_ is the differential hydrogen adsorption energy, which is defined as:

${\text{∆}\text{E}}_{\text{H*}}\text{=}\text{E}_{\text{(catalyst + n H)}\text{ }}\text{-}\text{ }\text{E}_{\left( \text{catalyst + }\left( \text{n - 1} \right)\text{H} \right)} \text{-}\text{ }\text{1/2}\text{E}_{\text{H}_{\text{2}}}$ (S4)

or the average chemisorption energy, which is defined as:

${\text{∆}\text{E}}_{\text{H*}}\text{=}\text{E}_{\text{(catalyst + n H)}\text{ }}\text{-}\text{ }\text{E}_{\mathrm{catalyst}} \text{-}\text{ }\text{n/2}\text{E}_{\text{H}_{\text{2}}}$ (S5)

Where $\text{E}_{\text{(catalyst + n H) }}$, $\text{E}_{\mathrm{catalyst}}$, and $\text{E}_{\text{H}_{\text{2}}}$ correspond to the total energies of H adsorbed intermediates, the catalyst system without H atom and H_2_ (gas phase). *n* is the number of H atoms adsorbed on the catalyst. In general, hydrogen generation is divided into individual process and collective process. For the former, equation (S5) describes the production of hydrogen molecule one by one, which can be assessed by d-Δ*G*_H*_. Moreover, equation (S3) for the latter represents all hydrogen atoms on the surface are simultaneously converted to molecules, which can be expressed by a-Δ*G*_H*_.

The exchange current *i*_0_ is based on Nørskov’s theory^[^[^4^](#_ENREF_4)^]^. If ΔG_*H_ ≤ 0, the following equation is used to calculate the exchange current at pH= 0:

$\text{i}_{\text{0}}\text{=-e}\text{k}_{\text{0}}\text{ }\text{/}\text{ }\text{[1+exp(}{\text{-∆}\text{G}}_{H*}\text{/}\text{k}_{\text{B}}\text{T})]$ (S6)

For another case if the Δ*G*_*H_ > 0, the *i*_0_ is calculated by using the following expression at pH= 0:

$\text{i}_{\text{0}}\text{=-e}\text{k}_{\text{0}}\text{ }\text{/}\text{ }\text{[1+exp(}{\text{∆}\text{G}}_{H*}\text{/}\text{k}_{\text{B}}\text{T})]$ (S7)

where *k*_0_ is the rate constant. As there are no experimental data available, *k*_0_ is set to 1. k_B_ is the Boltzmann constant (*k*_B_= 1.380649 × 10^−23^ J/K) and *T* is the temperature (*T*=298.15 K).

According to the reaction barrier and frequency analysis, the reaction rate constant (k) was defined by equation (S9) based on the Eyring transition state theory with Wigner correction.

$k\left( s^{-1} \right)=[1+ \frac{1}{24}\left( \frac{hv^{\neq}}{k_{B}T} \right)^{2}]\frac{k_{B}T}{h}e^{\frac{-\Delta G_{\mathrm{TS}}^{\neq}}{k_{B}T}}$ (S8)

where $\text{-Δ}\text{G}_{\text{TS}}^{\text{≠}}$ is the Gibbs free energy barrier, and its unit is kcal/mol. $\text{ν}\text{≠}$ is the absolute value of imaginary frequency of TS. (h: Planck constant, k_B_: Boltzmann constant, *T*: 298.15 K).

The entire reaction process of ORR is as follows:

4H^+^ +O_2_ +4e^-^ → 2H_2_O (S9)

The following are the four protonation reaction steps of the ORR:

* + O_2_ (g) + H^+^ + e^-^ → *OOH (S10)

*OOH + H^+^ + e^-^ → *O + H_2_O (l) (S11)

*O + H^+^ + e^-^ → *OH (S12)

*OH + H^+^ + e^-^ → * + H_2_O (l) (S13)

Where * represents the adsorption site, and *OOH, *O, *OH are the adsorption intermediate.

The OER is regarded as the opposite of the ORR, and the following are the four electron reaction steps of the OER and at each step, a proton is injected into the electrolyte:

H_2_O (l) + * → *OH + e^-^ + H^+^ (S14)

*OH → *O + e^-^ + H^+^ (S15)

H_2_O (l) + *O → *OOH + e^-^ + H^+^ (S16)

*OOH → O_2_ (g) + e^-^ + H^+^ + * (S17)

We show the calculated Δ*E* of intermediates *OOH, *O and *OH defined as the reaction energies of the reactions by the computational hydrogen electrode (CHE) model proposed by Nørskov and co-workers^[^[^5^](#_ENREF_5)^]^.

2H_2_O (g) + * → *OOH + 1.5H_2_ (g) (S18)

H_2_O (g) + * → *O + H_2_ (g) (S19)

H_2_O (g) + * → *OH + 0.5H_2_ (g) (S20)

The adsorption free energy of adsorption intermediate under pH = 0 is calculated by the following equation (*U* =0):

$\text{Δ}\text{G}\text{ }\text{=Δ}\text{E}\text{ + ΔZPE – }\text{T}\text{Δ}\text{S}$ (S21)

where H_2_O and H_2_ are in the gas phase. Δ*E*, ΔZPE and Δ*S* correspond to the total energy difference, zero-point energy difference, and entropy difference between final states and initial states according to eqs (S19) - (S21), respectively.

The free energy changes of each step of ORR are ${\text{Δ}\text{G}}_{\text{1}}\text{ = }{\text{Δ}\text{G}}_{\text{*OOH }}\text{ - 4.92eV}$, ${\text{Δ}\text{G}}_{\text{2}}\text{= }{\text{Δ}\text{G}}_{\text{*O }}\text{ - }{\text{Δ}\text{G}}_{\text{*OOH }}$, ${\text{Δ}\text{G}}_{\text{3}}\text{= }{\text{Δ}\text{G}}_{\text{*OH }}\text{ - }{\text{Δ}\text{G}}_{\text{*O }}$, and ${\text{Δ}\text{G}}_{\text{4}}\text{= - }{\text{Δ}\text{G}}_{\text{*OH }}$. Correspondingly, OER process: ${\text{Δ}\text{G}}_{a}\text{= }{\text{Δ}\text{G}}_{\text{*OH}}$, ${\text{Δ}\text{G}}_{\text{b}}\text{=}{\text{Δ}\text{G}}_{\text{*O}}\text{ - }{\text{Δ}\text{G}}_{\text{*OH}}$, ${\text{Δ}\text{G}}_{c}\text{=}{\text{Δ}\text{G}}_{\text{*OOH}}\text{ - }{\text{Δ}\text{G}}_{\text{*O}}$, ${\text{Δ}\text{G}}_{\text{d}}\text{ = 4.92eV}{\text{- }\text{Δ}\text{G}}_{\text{*OOH }}$. The overpotential (*η*) of ORR and OER is defined as

$\text{η}\text{ORR}\text{ = max \{}{\text{Δ}\text{G}}_{\text{1}}\text{, }{\text{Δ}\text{G}}_{\text{2}}\text{,}{\text{Δ}\text{G}}_{\text{3}}\text{, }{\text{Δ}\text{G}}_{\text{4}}\text{\}/e + 1.23V}$ (S22)

$\text{η}\text{OER}\text{ = max \{}{\text{Δ}\text{G}}_{\text{a}}\text{, }{\text{Δ}\text{G}}_{b}\text{,}{\text{Δ}\text{G}}_{c}\text{, }{\text{Δ}\text{G}}_{d}\text{\}/e - 1.23V}$ (S23)

Table S1 The models by doping a Pt atom at the core (Ni_5_Pt_1_@M_32_), the center or the hexagonal (Ni_6_@Pt_1_M_31_) site of the surface in the core-shell nanocluster Ni_6_@M_32_ (M = Pd, Cu, Ag, and Au).

|  | Substitution site | | |
| --- | --- | --- | --- |
|  | Core | Center | Hex |
| M | Ni_5_Pt_1_@M_32_ | Ni_6_@Pt_1_M_31_ | |
| Pd | 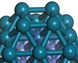 | 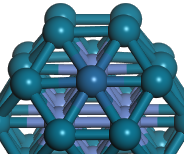 | 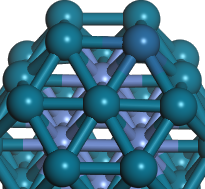 |
| Cu | 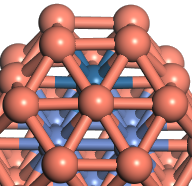 | 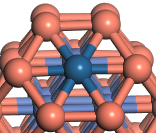 | 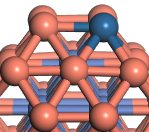 |
| Ag | 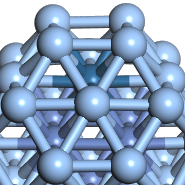 | 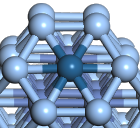 | 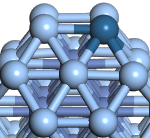 |
| Au | 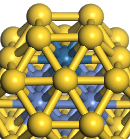 | 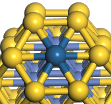 | 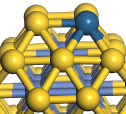 |

Table S2 Structural parameters of core-shell cluster catalysts

| System | Average bond length(Å) | | |
| --- | --- | --- | --- |
|  | shell-shell | shell-core | core-core |
| Pt_38_ | 2.75 | 2.75 | 2.75 |
| Ni_6_@Pt_32_ | 2.71 | 2.64 | 2.46 |
| Pd_38_ | 2.70 | 2.70 | 2.70 |
| Ni_6_@Pd_32_ | 2.68 | 2.62 | 2.42 |
| Ni_6_@Pt_1_Pd_31_ | 2.72 | 2.62 | 2.48 |
| Cu_38_ | 2.52 | 2.52 | 2.52 |
| Ni_6_@Cu_32_ | 2.47 | 2.51 | 2.50 |
| Ni_6_@Pt_1_Cu_31_ | 2.48 | 2.53 | 2.49 |
| Ag_38_ | 2.85 | 2.85 | 2.85 |
| Ni_6_@Ag_32_ | 2.79 | 2.82 | 2.57 |
| Ni_6_@Pt_1_Ag_31_ | 2.81 | 2.75 | 2.48 |
| Au_38_ | 2.84 | 2.84 | 2.84 |
| Ni_6_@Au_32_ | 2.79 | 2.78 | 2.46 |
| Ni_6_@Pt_1_Au_31_ | 2.79 | 2.77 | 2.47 |

Table S3 The optimal |d−Δ*G*_H*_| and |a−Δ*G*_H*_| and log(*i*_0_) values of Ni_6_@M_32_ (M= Pt, Pd, Cu, Ag, Au) and Ni_6_@Pt_1_M_31_ (M= Pd, Cu, Ag, Au) and corresponding Bader charge of hydrogen atom

| System | \| d-Δ*G*_H*_\| (eV) | \| a-Δ*G*_H*_ \| (eV) | exchange current density (log(*i*_0_))(A/cm^2^) | Bader charge of H atom (e) |
| --- | --- | --- | --- | --- |
| 6H-Ni_6_@Pt_32_ | 0.16 | 0.48 | -2.68 | -0.01 |
| 6H-Ni_6_@Pd_32_ | 0.09 | 0.33 | -1.54 | -0.12 |
| 1H-Ni_6_@Cu_32_ | 0.01 | 0.01 | -0.40 | -0.29 |
| 3H-Ni_6_@Ag_32_ | 0.01 | 0.002 | -0.40 | -0.22 |
| 1H-Ni_6_@Au_32_ | 0.002 | 0.002 | -0.32 | -0.30 |
| 6H-Ni_6_@Pt_1_Pd_31_ | 0.10 | 0.19 | -1.67 | -0.12 |
| 4H-Ni_6_@Pt_1_Cu_31_ | 0.004 | 0.006 | -0.34 | -0.16 |
| 1H-Ni_6_@Pt_1_Ag_31_ | 0.16 | 0.16 | -2.63 | -0.06 |
| 1H-Ni_6_@Pt_1_Au_31_ | 0.007 | 0.007 | -0.36 | -0.23 |

Table S4 The most stable adsorption sites and corresponding adsorption free energies of O, OH and OOH intermediates on the catalysts

| System | *O | | *OH | | *OOH | |
| --- | --- | --- | --- | --- | --- | --- |
|  | Site | Δ*G*_ad_/eV | Site | Δ*G*_ad_/eV | Site | Δ*G*_ad_/eV |
| Pt (111) | f | 1.30 | f | 0.42 | f | 3.27 |
| Pt_38_ | b1 | 1.24 | b1 | 0.50 | t2 | 3.81 |
| Ni_6_@Pt_32_ | b2 | 1.32 | t1 | 0.75 | t2 | 3.71 |
| Ni_6_@Pd_32_ | f1 | 1.50 | b1 | 0.19 | b3 | 3.54 |
| Ni_6_@Cu_32_ | f1 | 1.01 | b2 | -0.69 | b2 | 2.77 |
| Ni_6_@Ag_32_ | f1 | 1.59 | b2 | -0.57 | b2 | 2.89 |
| Ni_6_@Au_32_ | f1 | 2.10 | b2 | -0.21 | b3 | 3.81 |
| Ni_6_@Pt_1_Pd_31_ | f1 | 1.86 | f1 | 0.93 | b | 4.46 |
| Ni_6_@Pt_1_Cu_31_ | f1 | 1.39 | t | 0.40 | b | 4.22 |
| Ni_6_@Pt_1_Ag_31_ | f1 | 2.22 | b | 1.12 | b | 3.36 |
| Ni_6_@Pt_1_Au_31_ | f1 | 2.91 | t | 1.69 | t | 4.75 |

Table S5 The reaction free energy corresponding to the four-electron step of the ORR reaction on the catalyst and the overpotential of the ORR and OER reactions

| System | Δ*G*_1_ | Δ*G*_2_ | Δ*G*_3_ | Δ*G*_4_ | *η*^ORR^ | *η*^OER^ |
| --- | --- | --- | --- | --- | --- | --- |
| Pt (111) | -1.65 | -1.97 | -0.88 | -0.42 | 0.81 | 0.74 |
| Pt_38_ | -1.11 | -2.57 | -0.74 | -0.50 | 0.73 | 1.34 |
| Ni_6_@Pt_32_ | -1.21 | -2.40 | -0.57 | -0.75 | 0.66 | 1.17 |
| Ni_6_@Pd_32_ | -1.38 | -2.04 | -1.31 | -0.19 | 1.04 | 0.81 |
| Ni_6_@Cu_32_ | -2.15 | -1.76 | -1.70 | 0.69 | 1.92 | 0.92 |
| Ni_6_@Ag_32_ | -2.03 | -1.30 | -2.16 | 0.57 | 1.80 | 0.93 |
| Ni_6_@Au_32_ | -1.11 | -1.71 | -2.31 | 0.21 | 1.44 | 1.08 |
| Ni_6_@Pt_1_Pd_31_ | -0.46 | -2.59 | -0.94 | -0.93 | 0.77 | 1.36 |
| Ni_6_@Pt_1_Cu_31_ | -0.69 | -2.84 | -0.99 | -0.40 | 0.83 | 1.61 |
| Ni_6_@Pt_1_Ag_31_ | -1.56 | -1.13 | -1.11 | -1.12 | 0.12 | 0.33 |
| Ni_6_@Pt_1_Au_31_ | -0.17 | -1.84 | -1.22 | -1.69 | 1.06 | 0.61 |
| RuO_2_ |  |  |  |  |  | 0.37^[^[^6^](#_ENREF_6)^]^ |
| IrO_2_ |  |  |  |  |  | 0.56^[^[^6^](#_ENREF_6)^]^ |

Table S6 The average binding energy of Ni_6_@M_32_ (M= Ni, Pt, Pd, Cu, Ag, Au)

| System | *E*_b_(eV) | System | *E*_b_(eV) |
| --- | --- | --- | --- |
| Ni_6_@Pt_32_ | 4.53 | Ni_6_@Au_32_ | 2.96 |
| Ni_6_@Pd_32_ | 4.36 | Ni_6_@Ag_32_ | 2.52 |
| Ni_6_@Ni_32_ | 4.10 | Ni_6_@Cu_32_ | 0.25 |

Table S7 The formation energy of Ni_6_@Pt_1_M_31_ (M= Ni, Pd, Cu, Ag, Au)

| System | *E*_f_(eV) | System | *E*_f_(eV) |
| --- | --- | --- | --- |
| Ni_6_@Pt_1_Pt_31_ | 0 | Ni_6_@Pt_1_Au_31_ | 0.33 |
| Ni_6_@Pt_1_Pd_31_ | -0.56 | Ni_6_@Pt_1_Ag_31_ | -0.51 |
| Ni_6_@Pt_1_Ni_31_ | -0.30 | Ni_6_@Pt_1_Cu_31_ | -0.08 |

Table S8 Alteration in energy accompanying the migration of two hydrogen atoms from distinct adsorption sites (either on the same or different facets) on Ni_6_@Cu_32_ to a unified adsorption configuration

| H adsorption site | *E*_IS_(eV) | *E*_FS_(eV) | Δ*E*(eV) |
| --- | --- | --- | --- |
| Same facet | -130. 72 | -130.62 | 0.10 |
| Different facets | -130. 84 | -130.62 | 0.22 |


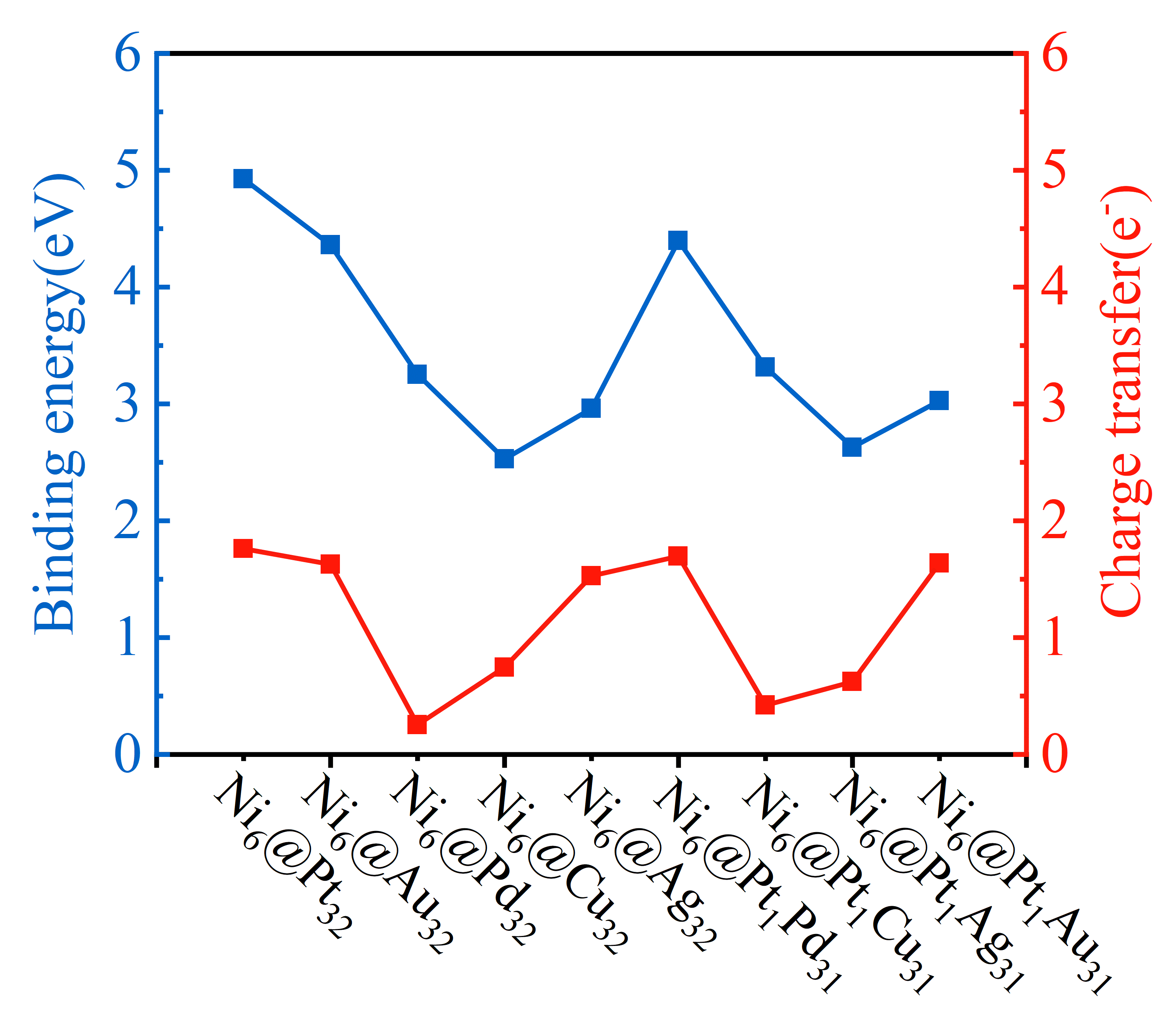


Figure S1 The relationship between the calculated average binding energy of catalysts and the amount of charge transfer between the core and shell (red represents the amount of Bader charge transfer, blue represents the average binding energy)


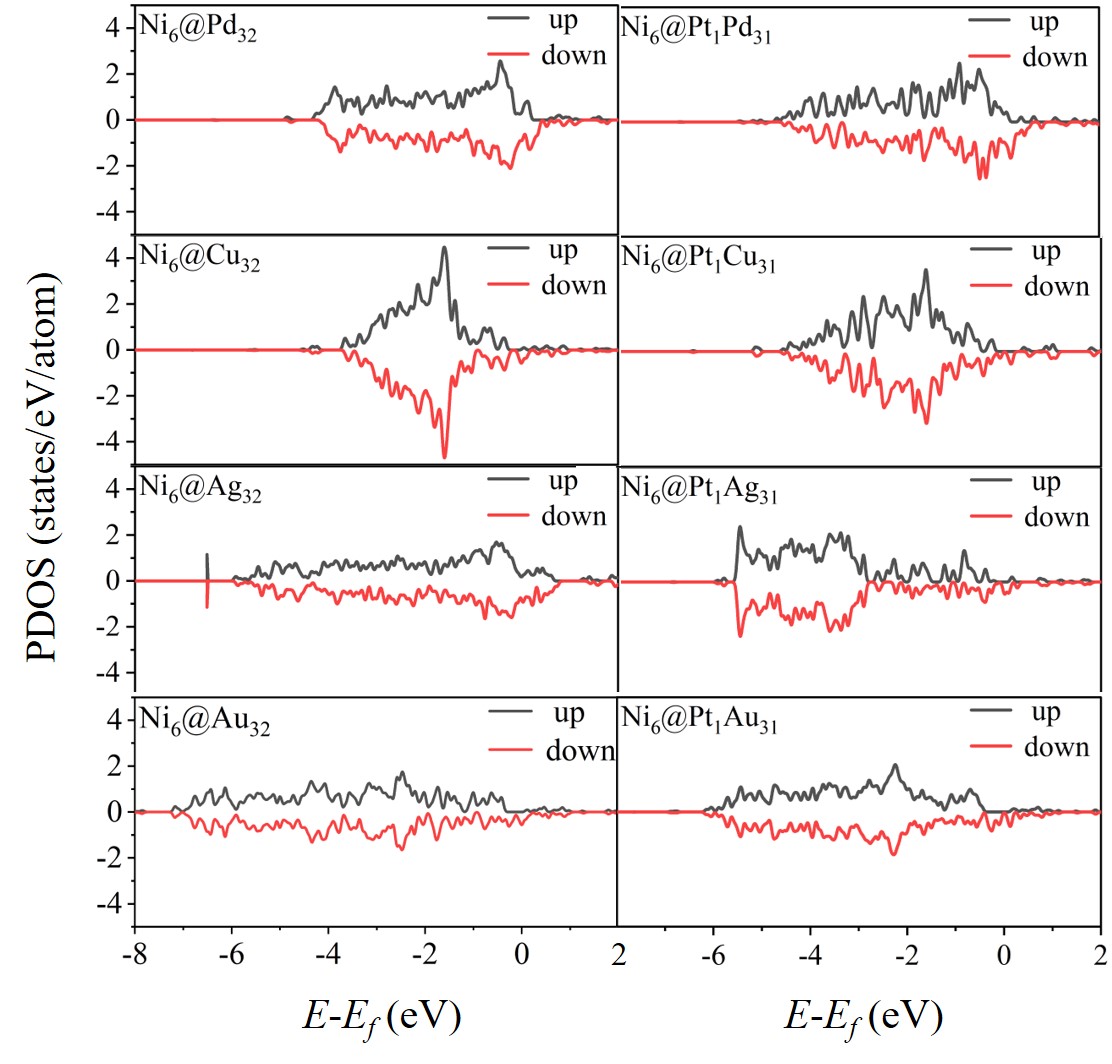


Figure S2 Density of states projected onto the d states of Ni_6_@M_32_ clusters (left) and Ni_6_@Pt_1_M_31_ (M=Pd, Cu, Ag, Au) clusters (right).


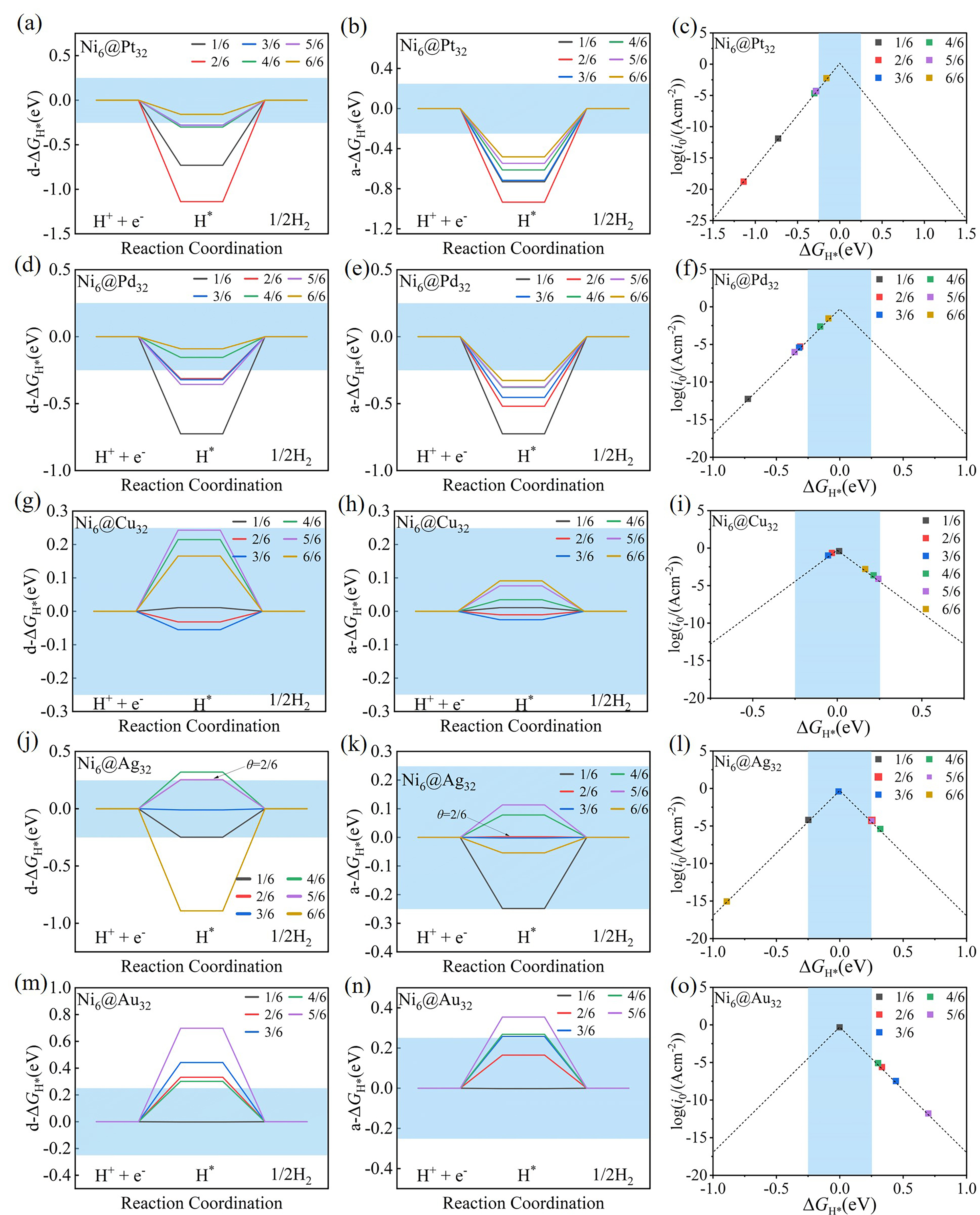


**Figure S3** The differential Gibbs free energy profiles (d−Δ*G*_H*_) over the Ni_6_@M_32_ (M = Pt, Pd, Cu, Ag, Au) clusters (a, d, g, j, m), the average Gibbs free energy profiles (a−Δ*G*_H*_) (b, e, h, k, n), and the volcano plot of *i*_0_ as a function of d-Δ*G*_H*_ at the best H coverage (c, f, i, l, o) and the highlight in blue denotes the free energy window of ±0.25 eV.


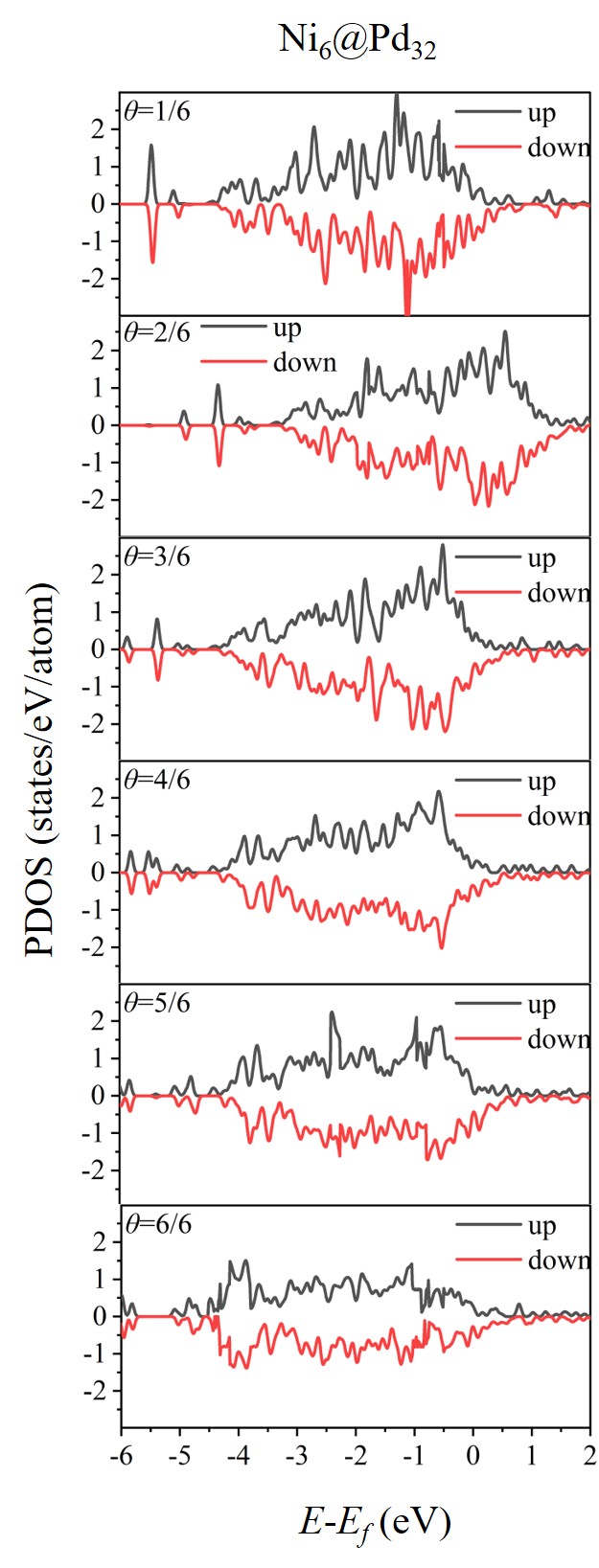

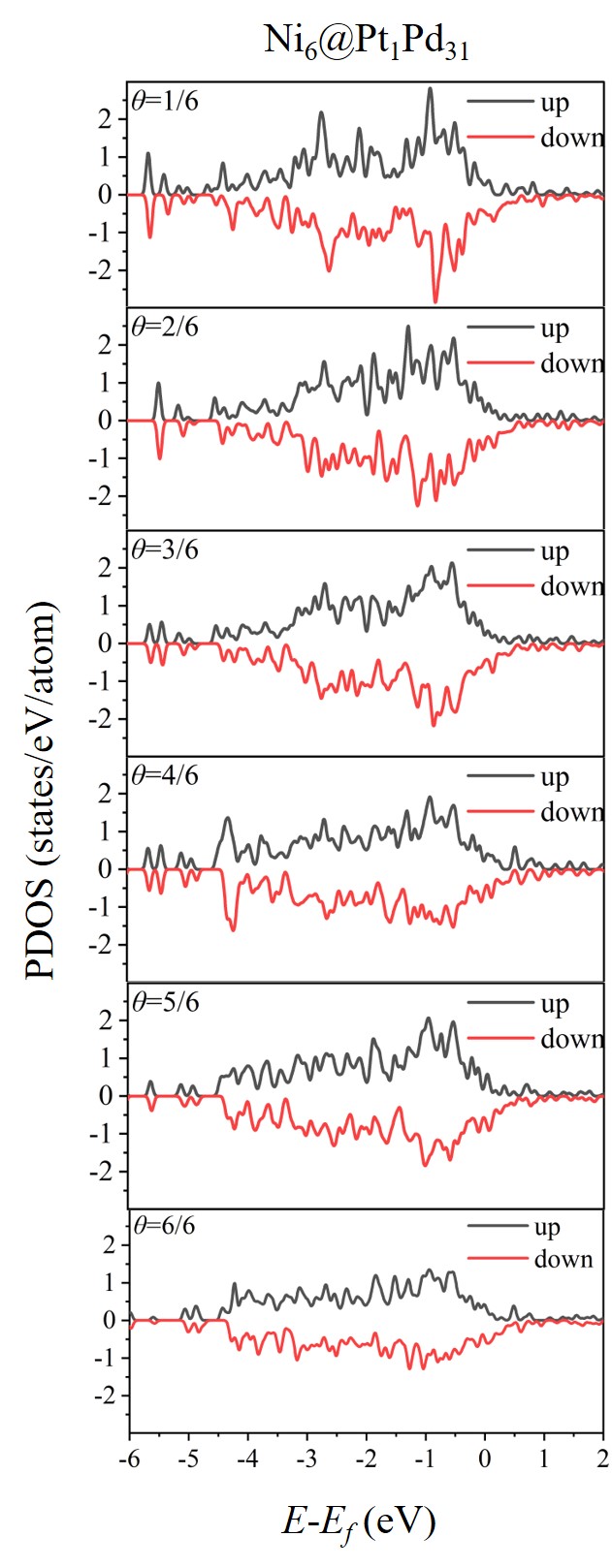


Figure S4 Density of states projected onto the d states of adsorption sites of Ni_6_@Pd_32_ clusters (left) and Ni_6_@Pt_1_Pd_31_ clusters (right) under different hydrogen coverages.


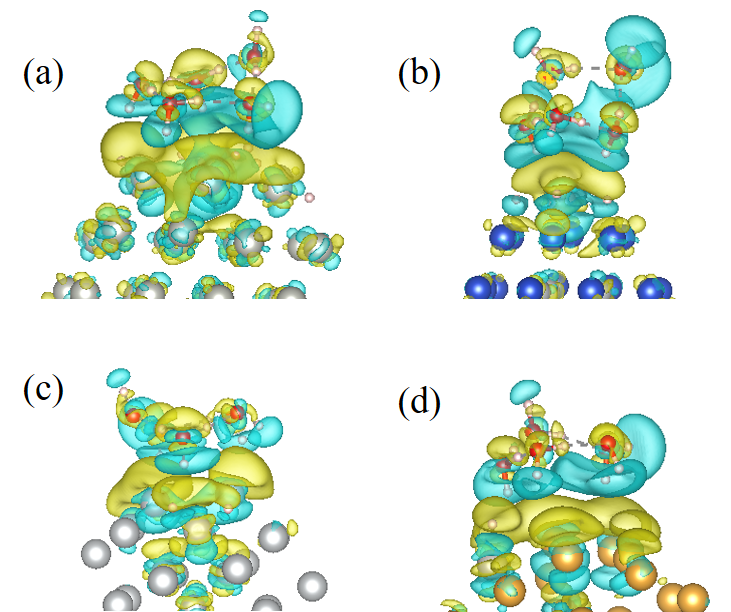


Figure S5 Insight into the electron density disparity at the solid-liquid interface in the initial state (Heyrovsky) is presented for the catalysts (a) 6H-Ni_6_@Pt_1_Pd_31_, (b) 4H-Ni_6_@Pt_1_Cu_31_, (c) 2H-Ni_6_@Pt_1_Ag_31_ and (d) 2H-Ni_6_@Pt_1_Au_31_. Regions of electron depletion and electron accumulation are depicted in cyan and light yellow, respectively.


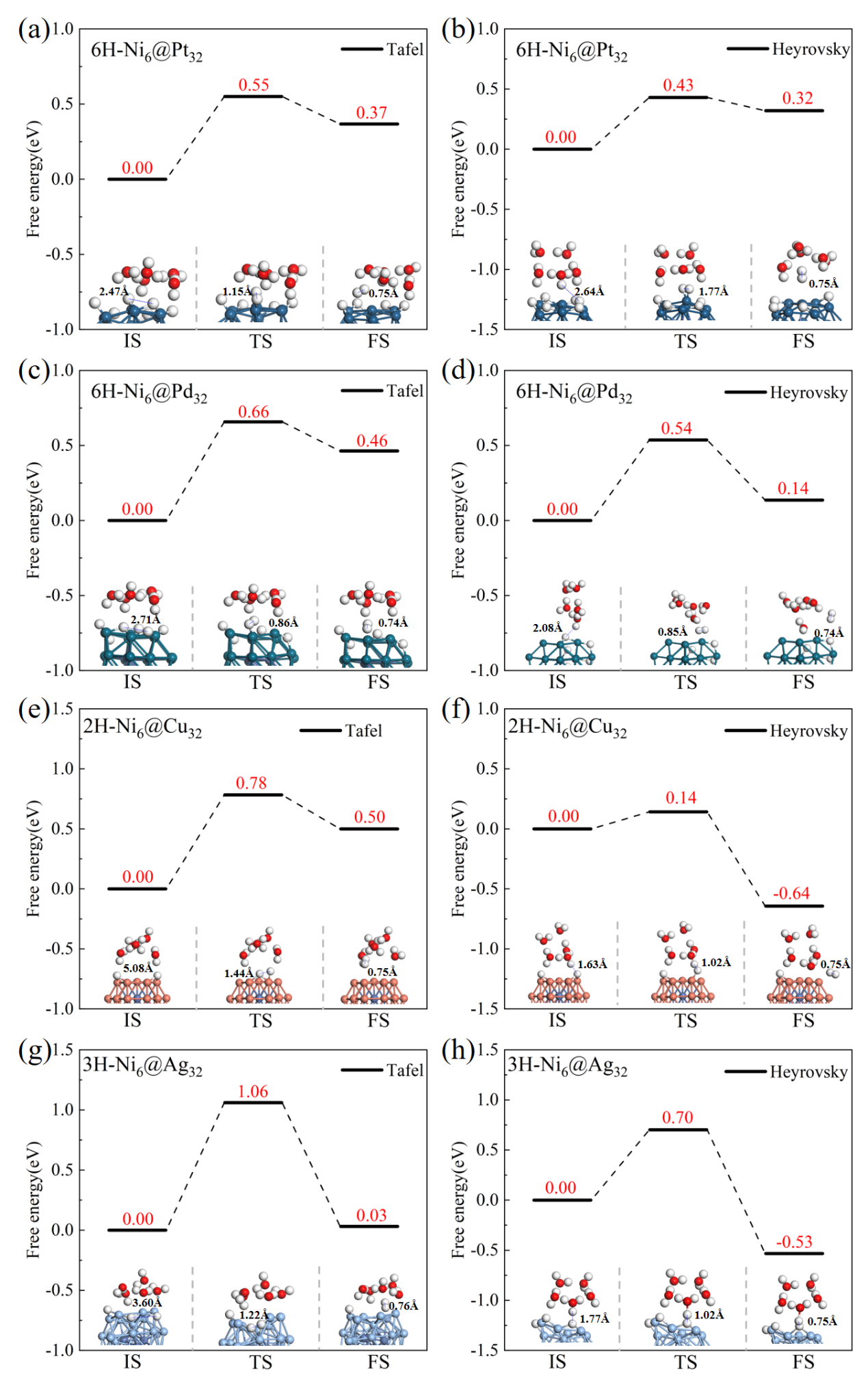


Figure S6 The potential energy profiles of the HER by Volmer–Tafel process and Volmer–Heyrovsky process at the optimal coverage of H. (a-b) Ni_6_@Pt_32_, (c-d) Ni_6_@Pd_32_, (e-f) Ni_6_@Cu_32_, (g-h) Ni_6_@Ag_32_, respectively


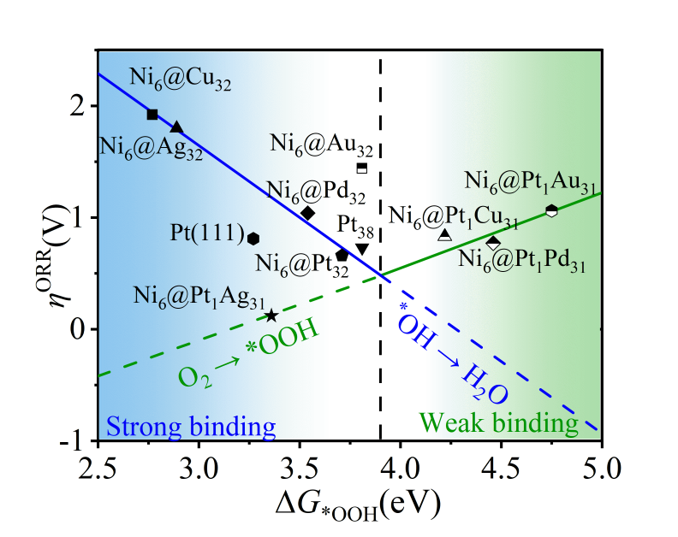


Figure S7 The linear relationship between ORR overpotential and *OOH adsorption free energy of catalysts according to four-electron step mechanism (ORR volcano plot)

**References:**

[1] Gennero de Chialvo MR, Chialvo AC. Kinetics of Hydrogen Evolution Reaction with Frumkin Adsorption: Re-Examination of the Volmer–Heyrovsky and Volmer–Tafel Routes[J]. Electrochimica Acta*,* 1998, 44(5): 841-851.

[2] de Chialvo MRG, Chialvo AC. Hydrogen Evolution Reaction: Analysis of the Volmer-Heyrovsky-Tafel Mechanism with a Generalized Adsorption Model[J]. Eelectroanal Chem*,* 1994, 372(1-2): 209-223.

[3] Gopalakrishnan D, Lee A, Thangavel NK, Reddy Arava LM. Facile Synthesis of Electrocatalytically Active NbS_2_ Nanoflakes for an Enhanced Hydrogen Evolution Reaction (HER)[J]. Sustain Energ Fuels*,* 2018, 2(1): 96-102.

[4] Nørskov JK, Bligaard T, Logadottir A, Kitchin JR, Chen JG, Pandelov S, Stimming U. Trends in the Exchange Current for Hydrogen Evolution[J]. J Electrochem Soc*,* 2005, 152(3).

[5] Nørskov JK, Rossmeisl J, Logadottir A, Lindqvist L, Kitchin JR, Bligaard T, Jónsson H. Origin of the Overpotential for Oxygen Reduction at a Fuel-Cell Cathode[J]. J. Phys. Chem. B*,*2004, 108(46): 17886-17892.

[6] Rossmeisl J, Qu ZW, Zhu H, Kroes GJ, Nørskov JK. Electrolysis of Water on Oxide Surfaces[J]. Electroanal Chem*,* 2007, 607(1-2): 83-89.
